# Optimal glycemic control in neurocritical care patients: a systematic review and meta-analysis

**DOI:** 10.1186/cc11812

**Published:** 2012-10-22

**Authors:** Andreas H Kramer, Derek J Roberts, David A Zygun

**Affiliations:** 1 Department of Critical Care Medicine, University of Calgary, ICU Administration - Ground Floor, McCaig Tower, 3134 Hospital Dr NW, Calgary, AB T2N 2T9, Canada; 2 Department of Clinical Neurosciences, University of Calgary, Room 1995 - Foothills Hospital, 1403 29th Street NW, Calgary, AB T2N 2T9, Canada; 3 Department of Community Health Sciences, University of Calgary, Faculty of Medicine - 3rd Floor TRW Building, 3280 Hospital Dr NW, Calgary, AB T2N 2T9, Canada; 4 Department of Surgery, University of Calgary, North Tower 10th Floor - Foothills Hospital, 1403 29th Street NW, Calgary, AB T2N 2T9, Canada

## Abstract

**Introduction:**

Hyper- and hypoglycemia are strongly associated with adverse outcomes in critical care. Neurologically injured patients are a unique subgroup, where optimal glycemic targets may differ, such that the findings of clinical trials involving heterogeneous critically ill patients may not apply.

**Methods:**

We performed a systematic review and meta-analysis of randomized controlled trials (RCTs) comparing intensive insulin therapy with conventional glycemic control among patients with traumatic brain injury, ischemic or hemorrhagic stroke, anoxic encephalopathy, central nervous system infections or spinal cord injury.

**Results:**

Sixteen RCTs, involving 1248 neurocritical care patients, were included. Glycemic targets with intensive insulin ranged from 70-140 mg/dl (3.9-7.8 mmol/L), while conventional protocols aimed to keep glucose levels below 144-300 mg/dl (8.0-16.7 mmol/L). Tight glycemic control had no impact on mortality (RR 0.99; 95% CI 0.83-1.17; p = 0.88), but did result in fewer unfavorable neurological outcomes (RR 0.91; 95% CI 0.84-1.00; p = 0.04). However, improved outcomes were only observed when glucose levels in the conventional glycemic control group were permitted to be relatively high [threshold for insulin administration > 200 mg/dl (> 11.1 mmol/L)], but not with more intermediate glycemic targets [threshold for insulin administration 140-180 mg/dl (7.8-10.0 mmol/L)]. Hypoglycemia was far more common with intensive therapy (RR 3.10; 95% CI 1.54-6.23; p = 0.002), but there was a large degree of heterogeneity in the results of individual trials (Q = 47.9; p<0.0001; I^2 ^= 75%). Mortality was non-significantly higher with intensive insulin in studies where the proportion of patients developing hypoglycemia was large (> 33%) (RR 1.17; 95% CI 0.79-1.75; p = 0.44).

**Conclusions:**

Intensive insulin therapy significantly increases the risk of hypoglycemia and does not influence mortality among neurocritical care patients. Very loose glucose control is associated with worse neurological recovery and should be avoided. These results suggest that intermediate glycemic goals may be most appropriate.

## Introduction

A key paradigm in the care of patients with acute brain and spinal cord injury is prevention of physiological abnormalities that may contribute to secondary neurological damage. Hyperglycemia is common in critically ill patients, and has been associated with worsened outcomes in the setting of traumatic brain injury (TBI) [1-9], aneurysmal subarachnoid hemorrhage (SAH) [10-19], spontaneous intracerebral hemorrhage (ICH) [20-26], ischemic stroke [27-35] and anoxic brain injury [36-38]. The mechanisms whereby hyperglycemia could be harmful are complex. Contributing factors may include free radical formation and oxidative injury, activation of N-methyl-D-aspartate receptors, raised intracellular calcium, triggering of inflammatory and apoptotic pathways, and alterations in lactate metabolism with reduced tissue pH [39]. Despite these observations, it remains unclear from human studies whether hyperglycemia is simply a marker for a greater severity of neurological damage or truly contributes to secondary injury in a causative fashion. Hypoglycemia may also be deleterious, since neurocritical care patients are dependent on sufficient glucose as an energy source for the central nervous system (CNS) [40,41]. Even moderate reductions in serum glucose can result in pronounced neuroglycopenia [42-44].

Numerous randomized controlled trials (RCTs) have assessed the efficacy and safety of intensive insulin therapy and tight glycemic control regimens in the care of critically ill patients. Despite initial enthusiasm based on the results of single-center RCTs [45,46], more recent multi-center RCTs have been unable to confirm any benefit, and have even suggested harm [47-49]. Similarly, meta-analyses have not demonstrated a reduction in mortality with tight versus conventional glycemic control [50-53].

Neurocritical care patients are a unique subgroup, in which the association between hyperglycemia and adverse outcomes in observational studies has been particularly strong. Although RCTs of tight glycemic control in critically ill patients have focused largely on mortality as the primary outcome, functional recovery is an especially meaningful endpoint in the neurologically injured. Even if an intervention does not impact on mortality, it may still be efficacious at improving functional and cognitive outcomes among survivors. Thus, the findings of RCTs involving heterogeneous populations of critically ill patients may not necessarily apply.

Some meta-analyses have pooled results in specific subgroups of brain-injured patients [54-56]. However, results from several RCTs were not included in these reviews. A comprehensive overview of all clinical trials involving neurocritical care patients has never been performed, and the optimal approach to glycemic control remains largely unknown. Therefore, we performed a systematic review and meta-analysis to assess whether tight glycemic control reduces mortality and improves outcomes in neurocritical care patients. We also conducted stratified analyses and meta-regression in an attempt to determine whether particular clinical or study-design characteristics influence the relationship between tight glycemic control and patient outcomes.

## Materials and methods

A written protocol, with a pre-specified analysis plan, was developed prior to study initiation in accordance with the Preferred Reporting Items for Systematic Reviews and Meta-analysis (PRISMA) statement [57].

### Search strategy

Using the OVID interface, we conducted unrestricted searches in MEDLINE, EMBASE, the Cochrane Central Register of Controlled Trials (CENTRAL) and the Cochrane Database of Systematic Reviews from their inception date until the first week of November 2011. To identify RCTs involving neurocritical care patients, the Boolean operator AND was used to combine three search concepts: intensive glycemic control, neurocritical care (defined below) and clinical trials. These concepts were created using a combination of Medical Subject Heading (MeSH) terms and keywords, and were combined using the Boolean operator OR (Additional file 1, Appendix).

A separate search was performed to identify clinical trials involving general critical care patients with heterogeneous diagnostic categories that were cared for in multi-system ICUs. Four published meta-analyses were used to identify relevant manuscripts [50-53], and the search strategy from one of these was repeated from March 2008 to November 2011 [51]. The manuscripts of retrieved studies were reviewed to determine if separate results were reported specifically for neurocritical care patients. We also searched the references of included RCTs and previous systematic reviews relating to intensive insulin therapy.

### Study selection

Article selection was performed in two sequential steps. First, one investigator (AHK) screened the title, abstract and keywords of all records retrieved using the search strategy. This stage was intended to be inclusive, and identified all RCTs involving hospitalized patients that compared at least two regimens of insulin administration or glycemic control. Second, the resultant, shorter list was reviewed independently and in duplicate by two investigators (AHK, and DJR).

Studies were considered eligible based on the following inclusion criteria: (1) study design (RCTs only); (2) target population (adults with at least one of the following conditions: TBI, SAH, ICH, ischemic stroke, anoxic injury, spinal cord injury or CNS infection); (3) intervention (comparing an intensive glycemic control protocol with a conventional (less tight) strategy); and (4) outcome (documentation of at least one of the primary or secondary outcomes (see below) in the target population).

Studies were excluded if other aspects of care, besides glycemic control, differed between groups. Thus, RCTs assessing the efficacy of glucose-potassium-insulin (GKI) regimens were not eligible, but were included in a planned sensitivity analysis. For RCTs involving mixed populations, but not presenting separate data for neurocritical care patients, we included the pooled results only if >75% of patients had a neurocritical care diagnosis. Studies consisting largely of non-emergent, perioperative neurosurgical patients were excluded, since these patients did not have an acute neurological injury.

### Data abstraction and assessment for risk of bias

Independently and in duplicate, two investigators (AHK, and DJR) abstracted data in an unblinded fashion, using a standardized form [58]. A translator was consulted to assist with papers published in a foreign language. Risk of bias among included RCTs was assessed using the following criteria: adequacy of allocation concealment, blinding of subjects and clinicians to treatment groups, blinding during outcome adjudication (for studies reporting neurological outcomes in addition to mortality), use of an intention-to-treat analysis, loss to follow-up, and baseline differences in important prognostic variables. In each case, we also assigned a Jadad score, which grades studies' descriptions of randomization (two points), blinding (two points) and attrition information (one point) [59]. Studies with an appropriate randomization strategy that prevented investigators or clinicians from predicting subsequent treatment allocation were considered to have adequate concealment [60]. For subsequent analyses, we categorized studies as having a relatively lower risk of bias if the Jadad score was >3 and there was adequate concealment of allocation. For studies reporting neurological outcomes, we also required outcome adjudication to have been performed in a blinded fashion.

Primary outcomes included: (1) 6-month mortality; if this was not specifically presented, we used the available time frame closest to 6 months, and (2) poor neurological recovery, as defined in individual studies. If a full range of outcomes was presented, we considered a Glasgow Outcome Scale (GOS) score of 1 to 3 (death, vegetative state or severe disability), a modified Rankin Scale (mRS) score of 4 to 6 (moderately severe disability, severe disability, death) or a cerebral performance category (CPC) of 3 to 5 (severe disability, coma or vegetative state, death) to represent poor outcomes.

Secondary outcomes, in each case using the definitions provided within individual studies, included the following: (1) hypoglycemia (if several definitions were provided, we utilized the threshold closest to 60 mg/dl); (2) nosocomial pneumonia; (3) other nosocomial infections.

### Data synthesis

Studies were pooled using Comprehensive Meta-Analysis (version 2.0, Biostat Inc., Englewood, NJ, USA). The risk ratio was chosen as the summary measure of association. Random effects models were used to pool risk ratios across studies and secondary analyses were performed using fixed effects models. Statistical heterogeneity was assessed with the *I*^2 ^statistic and *Q*-test (with a *P*-value < 0.10 considered significant) [61].

Potential reasons for variability in study results were anticipated in advance, and explored using pre-planned random effects meta-regression, in which patients were pooled *a priori *according to the following factors: glycemic targets in the control group (defined as loose if insulin was only initiated for glucose concentrations >200 mg/dl, and moderate if insulin was initiated for lower glucose concentrations); incidence of hypoglycemia (studies were dichotomized based on the median incidence, and the two groups were then compared); diagnosis (subgroups of studies involving patients with TBI or stroke were assessed separately); risk of bias (higher vs. lower, as defined above); and duration of intensive glycemic control (> 72 hours vs. <72 hours). We also planned sensitivity analyses with inclusion of studies involving GKI regimens or perioperative patients.

## Results

### Selection of studies

**Figure 1:**
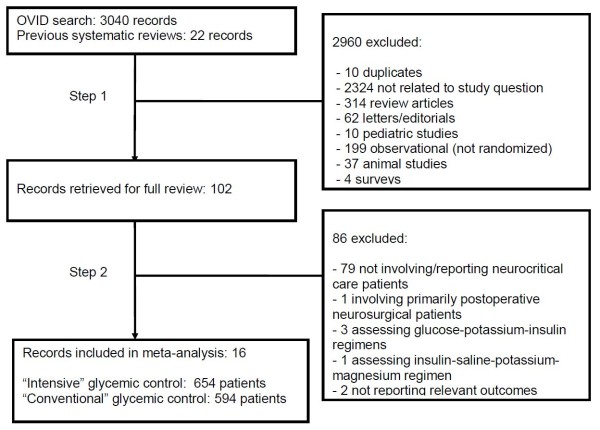
**Selection of randomized controlled trials comparing intensive and conventional glycemic control protocols in neurocritical care patients**.

Selection of studies is shown in Figure 1. Our initial search strategy identified 3,040 references. Of these, 90 involved a comparison of two insulin or glycemic control strategies in acute care patients. Another 22 papers, published prior to March 2008, were identified through previous meta-analyses of general critical care patients [50-53]. After removal of 10 duplicates, a list of 102 studies was reviewed in full during the second stage of article selection. Of these, 78 were excluded, leaving a total of 23 RCTs specifically assessing neurocritical care patients.

Of the 23 trials, one study involving perioperative neurosurgical patients was excluded because some of the data had already previously been published in two papers that were included in the meta-analysis. Moreover, the remaining patients primarily had brain tumors, which were treated with semi-elective surgery [62-64]. However, because this was a relatively large study, and the appropriateness of excluding elective neurosurgical patients is somewhat debatable, these results were incorporated into a secondary sensitivity analysis, from which the redundant data from the two other trials were removed [63,64]. Three RCTs involving patients with ischemic stroke used GKI regimens rather than only intensive insulin as their experimental treatment [65-67]. Another trial used an insulin-saline-potassium-magnesium infusion [68]. These four studies were excluded from the primary analysis, but their results were incorporated into a secondary analysis. Two additional RCTs were identified, but did not report any of our primary or secondary outcomes in the manuscript [69,70]. Thus, 16 studies, involving 1,248 patients (654 treated with intensive vs. 594 with conventional glycemic control), were retained for the determination of primary pooled outcomes [47,63,64,71-83].

### Characteristics of included studies

The target glucose concentration among patients treated with intensive insulin therapy was most often 80 to 110 mg/dl (4.4 to 6.1 mmol/L), but did vary slightly across RCTs, ranging from 70 to 150 mg/dl (3.9 to 8.3 mmol/L). Glucose goals were more variable in the conventional treatment groups. In the most extreme case, insulin therapy was only initiated when glucose levels exceeded 300 mg/dl (16.7 mmol/L), which was, at the time, consistent with AHA Guidelines for the management of ischemic stroke [76]. At the opposite extreme, one study had a conventional glucose target of 110 to 144 mg/dl (6.1 to 8.0 mmol/L) [77]. In most cases, insulin was only initiated in control patients when glucose levels exceeded 180 to 200 mg/dl. The duration of treatment varied from as short as 24 hours to the entire duration of the ICU admission. Definitions of hypoglycemia ranged from less than 40 to 80 mg/dl (2.2 to 4.4 mmol/L). The frequency of glucose monitoring for patients receiving intravenous (IV) insulin ranged from every 1 to 4 hours. Neurological outcomes were generally reported using the mRS, GOS or extended GOS. Relatively little information was provided on the provision of nutrition; in most cases tube feeding appeared to have been initiated as soon as possible to patients who could not eat (Table 1).

**Table 1:** Characteristics of studies comparing intensive and conventional glycemic control in neurocritical care patients

Author, year	Patients, number	Diagnosis	Intensive definition	Conventional definition	Duration of protocol	Definition of hypoglycemia	Definition of poor outcome	Timing of nutrition
Staszewski, 2011	50	IS	81-126 mg/dl (iv insulin)	< 180 mg/dl (sc insulin)	24 hours	< 60 mg/dl	mRS 3-6 (30 days)	Deferred 24 hours
Green, 2010	81	Mixed	80-110 mg/dl (iv insulin)	< 150 mg/dl (sc insulin)	ICU stay	< 60 mg/dl	mRS 3-6 (3 months)	EN within 24 hours
Coester, 2010	88	TBI	80-110 mg/dl	< 180 mg/dl	ICU stay	< 80 mg/dl	GOS 1-3 (6 months)	EN within 24-48 hours
Johnston, 2009	74	IS	70-110 mg/dl	< 200 mg/dl (loose) < 300 mg/dl (usual)	5 days	< 55 mg/dl	mRS 2-6 (3 months)	PO or EN ASAP
Azevedo, 2009	34	IS	< 140 mg/dl (IV insulin)	< 150 mg/dl (SC insulin)	NR	NR	eGOS (Hospital dc)	Not specified; carbohydrate restriction in controls
Meng, 2009	240	TBI	80-110 mg/dl	180-200 mg/dl	ICU stay	< 40 mg/dl	GOS 1-3 (6 months)	IV glucose for 24 hours then EN or PN
Yang, 2009	110	ICH, IS, SAH	80-150 mg/dl (IV insulin)	Treated with twice daily insulin 30/70	ICU stay	< 80 mg/dl	mRS 4-6 (time frame unclear)	Not specified
Bilotta, 2008	97	TBI	80-120 mg/dl	< 220 mg/dl	ICU stay	< 80 mg/dl	GOS 1-3 (6 months)	EN or PN ASAP
Kreisel, 2008	40	IS	80-110 mg/dl	< 200 mg/dl	5 days	< 60 mg/dl	RS > 2	Not specified
Arabi, 2008	94	TBI	80-110 mg/dl	180-200 mg/dl	ICU stay	< 40 mg/dl	NR	EN ASAP
Bruno, 2008	46	IS	90-130 mg/dl	< 200 mg/dl	72 hours	< 60 mg/dl	mRS 3-6 (3 months)	Not specified
Oksanen, 2007	90	AI	80-110 mg/dl	110-144 mg/dl	48 hours	< 55 mg/dl	NR	Not specified
Azevedo, 2007	48	Mixed	80-120 mg/dl	< 180 mg/dl	ICU stay	< 40 mg/dl	eGOS (3 months)	IV glucose for 48 hours then EN; carbohydrate restriction in controls
Bilotta, 2007	78	SAH	80-120 mg/dl	< 220 mg/dl	ICU stay	< 80 mg/dl	mRS 4-6 (6 months)	EN or PN ASAP
Walters, 2006	25	IS	90-144 mg/dl	< 270 mg/dl	48 hours	NR	NR	Deferred 48 hours
Van den Berghe, 2005	63	Mixed	80-110 mg/dl	< 200 mg/dl	ICU stay	<40 mg/dl	Karnofsky > 60 (12 months)	IV glucose for 24 hours then EN or PN

AI, anoxic brain injury; ASAP, as soon as possible; eGOS, extended GOS; EN, enteral nutrition; GCS, Glasgow Coma Scale; GOS, Glasgow Outcome Scale; ICH, intracerebral hemorrhage; IS, ischemic stroke; mRS, modified Ranking Scale; NR, not reported; PN, parenteral nutrition; RS, Rankin score; SAH, subarachnoid hemorrhage; TBI, traumatic brain injury.

The risk of bias varied across studies. In no study were clinicians blinded to glucose levels. For this reason, the Jadad score was never > 3. Most studies used an intention-to-treat analysis and loss to follow-up was relatively uncommon. Baseline characteristics among patients in the two groups were largely similar. Individuals adjudicating neurological outcomes were not always blinded with respect to the treatment group (Table 2).

**Table 2:** Risk of bias in studies comparing intensive and conventional glycemic control in neurocritical care patients

Author (year)	Concealed allocation	Description of random allocation method	Double-blind	ITT analysis	All patients accounted for	Major baseline differences	Blinded outcome adjudication	Jadad score
Staszewski, 2011	Unclear	No	No	Yes	Yes	Mean age higher in conventional group (87 vs. 68 yrs; NS)	Yes	2
Green, 2010	Adequate	No	No	Yes	7 patients lost	No	Yes	2
Coester, 2010	Adequate	No	No	No^†^	Yes	More poly-trauma, normal CT scans in control patients	Unclear	2
Johnston, 2009	Adequate	No	No	Yes	1 patient lost (incarcerated)	No	Yes	2
Azevedo, 2009	Unclear	No	No	Yes	No^‡^	Unclear	Unclear	1
Meng, 2009	Adequate	No	No	Yes	7 patients lost	No	Yes	2
Yang, 2009	Unclear	No	No	Yes	Yes	No	Unclear	2
Bilotta, 2008	Adequate	Yes	No	Yes	Yes	No	Yes	3
Kreisel, 2009	Adequate	Yes	No	Yes	3 patients lost	More males in intensive group	No	3
Arabi, 2008	Unclear	Yes	No	Yes	Yes	Unclear	Not relevant	3
Bruno, 2008	Adequate	No (although done by "data management center")	No	Yes	Yes	More patients with diabetes mellitus, treated with tPA in intensive group	Yes	2
Oksanen, 2007	Adequate	No (although done by "independent statistician"	No	Yes	Yes	More patients male in ITT groups. Lower MAP in ITT group.	Not relevant	2
Azevedo, 2007	Adequate	Yes	No	Yes	No*	No	No	2
Bilotta, 2007	Adequate	Yes	No	Yes	Yes	No	Yes	3
Walters, 2006	Unclear	No (although done by pharmacy using "standard algorithm")	No	Yes	Unclear	More patients with high HbA1C in treatment group	Not relevant	1
Van den Berghe, 2005	Adequate	No	No	Yes	Yes	More males, patients diabetes mellitus, malignancy, ICH, SAH in intensive group	Yes	2

^† ^Although authors stated they used intention-to-treat (ITT) analysis, description of patient flow suggests otherwise. Eight patients did not receive their allocated treatment; their results were not presented or analyzed (largely because unable to obtain consent after randomization); ^‡ ^20 patients randomized to conventional group; 6 died; functional outcome information only described for 12 (rather than 14); *numbers in Table 3 of manuscript do not account for all patients. NS, not significant; CT, computer tomography; ICH, intracerebral hemorrhage; MAP, mean arterial pressure; SAH, subarachnoid hemorrhage; tPA, tissue plasminogen activator.

### Effect of intensive glycemic control on mortality and poor neurological outcomes

**Figure 2:**
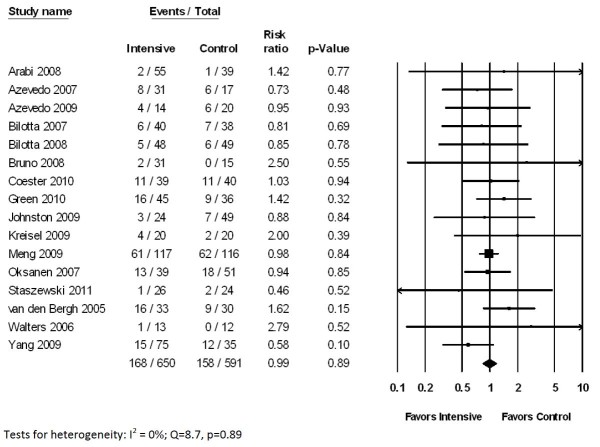
**Impact of intensive glycemic control on mortality in neurocritical care patients**.

There was no statistically significant difference in mortality between patients treated with intensive (26%) versus conventional glycemic targets (27%) (relative risk, RR 0.99, 95% CI 0.83 to 1.17, *P *= 0.89) (Figure 2). There was little heterogeneity in study results (*Q *= 8.7, *P *= 0.89; *I*^2 ^= 0%). Findings were consistent in five RCTs involving patients with TBI (RR 0.99, 95% CI 0.79 to 1.22, *P *= 0.89), six RCTs of patients with ischemic stroke (RR 1.10, 95% CI 0.57 to 2.12, *P *= 0.78), and nine RCTs of patients with any type of stroke (ischemic or hemorrhagic; RR 0.91, 95% CI 0.61 to 1.34, *P *= 0.63).

**Figure 3:**
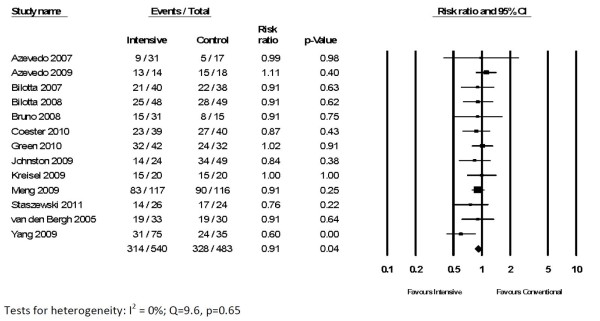
**Impact of intensive glycemic control on poor functional recovery in neurocritical care patients**.

In 13 RCTs reporting neurological recovery in 1,023 randomized patients, intensive glycemic control resulted in a lower risk of poor neurological outcomes (58% vs. 68%; RR 0.91, 95% CI 0.84 to 1.00, *P *= 0.04) (Figure 3). There was no significant heterogeneity (*Q *= 9.6, *P*= 0.65; *I*^2 ^= 0%). A comparable trend was observed in four RCTs involving 449 patients with TBI (RR 0.91, 95% CI 0.80 to 1.02, *P *= 0.11) and in eight RCTs involving 457 patients with either ischemic or hemorrhagic stroke (RR 0.90, 95% CI 0.77 to 1.05, *P *= 0.19). Among 241 patients specifically with ischemic stroke, intensive insulin had no clear effect (RR 0.97, 0.83 to 1.14, *P *= 0.71).

### Effect of intensive glycemic control on secondary outcomes

**Figure 4:**
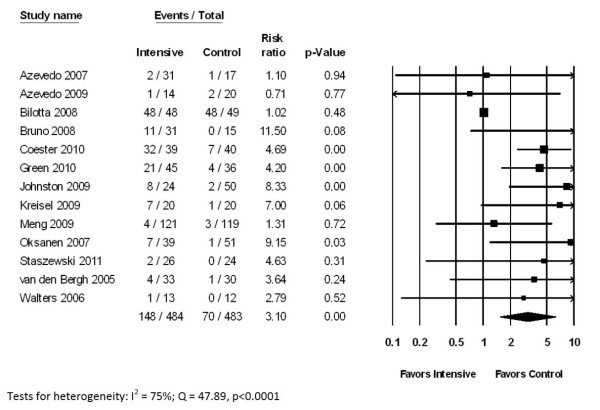
**Impact of intensive glycemic control on incidence of hypoglycemia in neurocritical care patients**.

Thirteen trials, involving 967 patients, reported the incidence of hypoglycemia. The proportion of patients treated with intensive insulin who developed hypoglycemia varied greatly between studies, ranging from 3 to 100%, with a median value of 18 to 33%. Although definitions varied, the incidence of hypoglycemia was markedly greater among patients treated with intensive insulin protocols (30% vs. 14%; RR 3.10, 95% CI 1.54 to 6.23, *P *= 0.002) (Figure 4). However, there was a large degree of heterogeneity between studies (*Q *= 47.9, *P *< 0.0001, *I*^2 ^= 75%).

Six RCTs reported the incidence of pneumonia. Intensive glycemic control did not have any protective effect (RR 1.04, 95% CI 0.82 to 1.32, *P *= 0.73). Mild to moderate heterogeneity between studies was observed (*Q *= 6.0, *P *= 0.31, *I*^2 ^= 17%). Other nosocomial infections were infrequently reported, such that we did not pool the results.

### Meta-regression & sensitivity analyses

Results of subgroup analysis and meta-regression are shown in Table 3. Of the 13 studies reporting the occurrence of neurological outcomes, eight used a control group where glycemic control could be considered, according to our *a priori *definition, to have been very loose, with insulin administered only if glucose was >200 mg/dl (11.1 mmol/L). Five studies used a design where even the control group received insulin to maintain glucose levels within a relatively narrow range, with a threshold for insulin administration of 144 to 180 mg/dl (8.0 to 10.0 mmol/L). An improvement in outcomes was only observed in the subgroup of studies where control group glucose levels were allowed to be relatively high (RR 0.88, 95% CI 0.79 to 0.98, *P *= 0.02), but not in those where there was a less extreme difference (RR 0.99, 95% CI 0.85 to 1.14, *P *= 0.84). The difference between these two categories of studies was statistically significant (*P *= 0.04).

**Table 3:** Subgroup analysis and meta-regression of studies assessing the efficacy of intensive glycemic control in neurocritical care patients

Comparison	Mortality			Poor neurological outcome		
	**Studies, number**	**Risk ratio (95% confidence intervals)**	***P*-value for comparison**	**Studies, number**	**Risk ratio (95% confidence intervals)**	***P*-value for comparison**

Control group						
Very loose	10	0.98 (0.80-1.20)		8	0.88 (0.79-0.98)	
Moderate	6	1.00 (0.72-1.39)	0.89	5	0.99 (0.85-1.14)	0.04
Hypoglycemia†						
Uncommon	7	1.00 (0.86-1.24)		5	0.94 (0.84-1.06)	
Common	6	1.17 (0.78-1.76)	0.72	6	0.94 (0.81-1.08)	0.94
Duration of tight control						
> 72 hours	12	0.99 (0.83-1.18)		11	0.92 (0.84-1.01)	
<72 hours	4	0.97 (0.56-1.67)	0.37	2	0.81 (0.57-1.15)	0.04
Risk of bias						
Higher	11	1.03 (0.86-1.24)		10	0.94 (0.85-1.04)	
Lower	4	1.00 (0.52-1.91)	0.76	3	0.94 (0.76-1.17)	0.17
Timing of nutrition						
As soon as possible	6	1.07 (0.73-1.57)		5	0.93 (0.80-1.08)	
Deferred > 24 hours	5	1.01 (0.81-1.26)	0.82	4	0.90 (0.79-1.03)	0.79
Definition						
Hypoglycemia	8	0.94 (0.67-1.31)		8	0.88 (0.77-1.00)	
56-80 mg/dl 55 mg/dl or below	6	1.01 (0.82-1.23)	0.72	4	0.91 (0.79-1.03)	0.72

^† ^As per *a priori *plan, patients were dichotomized based on the median prevalence of hypoglycemia across studies; common, hypoglycemia occurred in 33-100% of patients; uncommon, hypoglycemia occurred in 3 to 18% of patients.

As per our *a priori *plan, studies were dichotomized into those having a high (33 to 100%) or a low incidence (3 to 18%) of hypoglycemia. A non-significant increment in mortality was seen in studies where the incidence of hypoglycemia was high (RR 1.17, 95% CI 0.78 to 1.76, *P *= 0.44). However, this result did not differ statistically when compared with studies where the incidence of hypoglycemia was low.

Twelve studies assessed the efficacy of intensive insulin administered for more than 72 hours. In four studies, intensive insulin was used more briefly, for time intervals ranging from 24 to 72 hours. No differences in mortality were observed based on the duration of time that intensive insulin was administered. In 11 of the 12 studies using more prolonged regimens of intensive insulin, neurological outcomes were reported and there was a trend towards an improvement with intensive therapy (RR 0.92, 95% 0.84 to 1.01, *P *= 0.07).

Inclusion of the trial that involved postoperative neurosurgical patients (and exclusion of patients for whom there were redundant data) had little impact on the results [62-64]. On combining the studies assessing the impact of GKI or insulin-saline-potassium-magnesium infusions in ischemic stroke patients, there was no improvement in mortality (reported in three studies; RR 1.08, 95% CI 0.89 to 1.32, *P *= 0.43) or neurological recovery (reported in three studies; RR 1.02, 95% CI 0.94 to 1.10, *P *= 0.63). When these four RCTs were combined with all other RCTs, the improvement in functional outcomes associated with intensive glycemic control was no longer present (RR 0.95, 95% CI 0.89 to 1.01, *P *= 0.11).

Five studies deferred nutritional supplementation for 24 to 48 hours, of which three explicitly mentioned providing intravenous glucose during this time (Table 1). No difference in mortality or unfavourable outcomes was observed in comparison to RCTs where enteral nutrition was not delayed. Three RCTs explicitly mentioned providing intravenous glucose supplementation to patients who were not receiving any other nutrition; in contrast to most other studies, intensive insulin did not significantly increase the incidence of hypoglycemia in these trials (RR 1.64, 0.56 to 4.80, *P *= 0.37) [71,79,81].

We also assessed outcomes of studies based on the definition of hypoglycemia that was used. Eight RCTs defined hypoglycemia using a relatively high threshold of glucose ≤ 60 to 80 mg/dl (3.3 to 4.4 mmol/L) and six studies used a low threshold of glucose ≤ 40 to 55 mg/dl (2.2 to 3.1 mmol/Ll). There were no differences in mortality, neurological recovery or the incidence of hypoglycemia based on these thresholds.

### Publication bias

**Figure 5:**
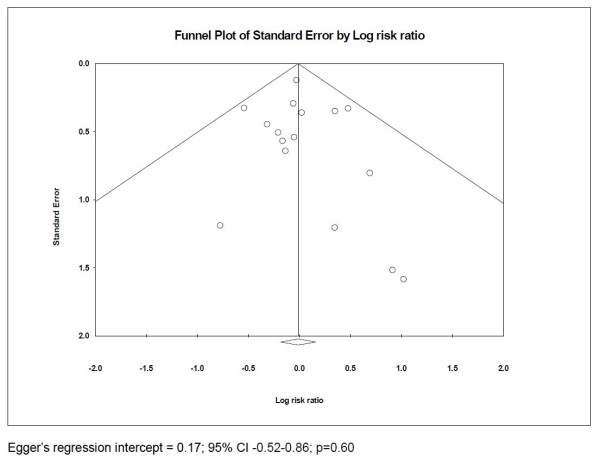
**Funnel plot showing standard error of studies assessing efficacy of intensive glycemic control in neurocritical care patients in relation to log of calculated risk ratio**.

Visual inspection of a funnel plot revealed relative symmetry, arguing against the presence of publication bias (Figure 5). Similarly, there was no evidence of publication bias using Egger's test (intercept 0.17, 95% CI -0.52 to 0.86 *P *= 0.60).

## Discussion

Our results provide the most contemporary and comprehensive overview of RCTs involving different glycemic control strategies in neurocritical care patients. Previous quantitative systematic reviews have been published [54,55], but they did not include multiple relevant publications [47,72-78,83], they were based in part on redundant data [62-64], and they did not perform stratified analyses and meta-regression in order to explain heterogeneity in RCT results.

Our findings suggest that intensive glycemic control does not reduce mortality among neurocritical care patients. This observation is consistent with the results of recent large, multi-center RCTs performed in critically ill patients with more heterogeneous, and not necessarily neurological, diagnostic categories [47-53].

In contrast, we did observe intensive glycemic control to reduce the occurrence of poor neurological outcomes. This finding was largely limited to the subgroup of studies where target glucose concentrations in the control group were very loose (insulin initiated only when glucose concentration exceeded 200 mg/dl). A benefit was not observed when intensive treatment was compared with more intermediate glycemic targets (110 to 180 mg/dl). This observation suggests that some of the benefit from intensive insulin may instead have been related to harm attributable to loose glucose control. Thus, glucose concentrations in excess of 180 mg/dl should be avoided in neurocritical care patients. This finding is consistent with a large number of animal experiments and human observational studies.

We found that the incidence of hypoglycemia was markedly increased by intensive insulin therapy. However, the rate of hypoglycemia varied greatly across RCTs. Patients treated with intensive treatment had somewhat higher mortality in studies where the incidence of hypoglycemia was high (>33%), although this result was not statistically significant. Hypoglycemia has been shown to be a strong predictor of mortality in critically ill patients [47,84]. In brain-injured patients, microdialysis studies have demonstrated that reductions in serum glucose concentration may produce profound neuroglycopenia, which in turn may contribute to metabolic distress and secondary brain injury [43,85-88]. Hypoglycemia may also help explain why the introduction of an intensive insulin protocol has been associated with worse outcomes at some centers [89].

One of the proposed complications of hyperglycemia is an increased vulnerability to nosocomial infections. Only a small proportion of studies involving neurocritical care patients reported infection rates. When the results were combined, we could not find any impact of glycemic control on the incidence of hospital-acquired pneumonia.

We did not identify one subgroup of neurocritical care patients in whom intensive insulin therapy was associated either with any particular benefit or harm. The relationship between tighter glycemic control and improved neurological recovery was, however, stronger among patients with TBI, ICH or SAH than it was for patients with ischemic stroke. This finding is consistent with the lack of benefit observed in RCTs assessing the efficacy of GKI infusions, all of which exclusively involved patients with ischemic stroke [65-67]. Our findings should not necessarily be applied to patients undergoing semi-elective neurosurgical procedures, such as resection of a brain tumor, since these were not included in the analysis.

We believe that RCTs are consistent with a U-shaped relationship between serum glucose concentration and neurological outcomes [43]. Both hypoglycemia and extreme hyperglycemia are likely to be harmful. Comparable observations have also been made in medical and surgical critical care patients [90]. The optimal glucose target for neurocritical care patients is likely to fall between 80 and 180 mg/dl (4.4 and 10.0 mmol/L). Given that RCTs suggest a relatively high incidence of hypoglycemia when clinicians attempt to maintain glucose levels between 80 and 110 mg/dl (4.4 to 6.1 mmol/L), we consider a more conservative approach to be most appropriate, for example, 110 to 180 mg/dl (6.1 to 10.0 mmol/L).

Some large RCTs involving heterogeneous populations of critically ill patients have not yet published results for their subgroup of neurological patients, and were therefore excluded from this analysis. Most importantly, the NICE-SUGAR trial included more than 6,000 critically ill patients [49]. The GLUCONTROL trial designated 142 of 1,078 patients (13%) as having a neurological diagnostic category, but did not provide results for this subgroup [91]. Another trial, involving 1,200 medical ICU patients, reported hospital mortality among 61 patients with neurologic conditions. Because the specific disorders were not described, it was unclear if these patients met our eligibility criteria [46]. We were unable to obtain this information from the authors. However, a sensitivity analysis performed with inclusion of these patients did not change our results (RR 0.99, 0.84 to 1.17, *P *= 0.90).

There are further limitations to this meta-analysis. Although there were many similarities to the methodology of the included RCTs, there was also some variability. This variability is especially reflected by the wide range of hypoglycemia (3 to 100%) among patients randomized to intensive insulin protocols. Any future RCTs of intensive insulin should therefore first carefully pilot their protocol to ensure that hypoglycemia can be minimized. There was some heterogeneity in the provision and reporting of nutritional supplementation, which may have influenced the results. Neurological outcomes reported in this meta-analysis were relatively crude; it remains possible that glycemic control could have a greater influence on more subtle neurocognitive or indices of quality of life. Finally, although we have clustered various neurocritical care conditions, there may be significant differences across disease states, or based on brain-injury severity, that may influence the pathophysiology and implications of hyperglycemia.

## Conclusions

In summary, a growing number of RCTs, involving many hundreds of patients, cumulatively demonstrate that intensive glycemic control does not reduce mortality in neurocritical care patients. A unique benefit in certain subgroups cannot be excluded, but no such trend was observed in our analysis. Very loose glycemic control with a target of > 180 mg/dl (10 mmol/L) appears to be harmful and should be avoided. Intensive control with target glucose of 80 to 110 mg/dl (4.4 to 6.1 mmol/L) greatly increases the risk of hypoglycemia. Thus, at present, the literature supports targeting more intermediate glucose levels.

## Key messages

• Intensive glycemic control does not appear to improve mortality in neurocritical care patients.

• Very loose glycemic control with insulin initiated only for glucose concentrations >200 mg/dl (11.1 mmol/L) is associated with poor neurological outcomes in neurocritical care patients, compared with either intensive insulin therapy with a target glucose concentration of 80 to 110 mg/dl (4.4 to 6.1 mmol/L), or more modest glycemic control with a target glucose concentration of 110 to 180 mg/dl (6.1 to 10.0 mmol/L).

• Intensive glycemic control greatly increases the risk of hypoglycemia in neurocritical care patients.

## Abbreviations

CNS: central nervous system; CPC: Cerebral Performance Category; GOS: Glasgow Outcome Scale; GKI: glucose potassium insulin; ICH: intracerebral hemorrhage; IV: intravenous; mRS: modified Rankin Scale; PRISMA: Preferred Reporting Items for Systematic Reviews and Meta-analysis; RCT: randomized controlled trial; RR: relative risk; SAH: subarachnoid hemorrhage; TBI: traumatic brain injury.

## Competing interests

The authors declare that they have no competing interests.

## Authors' contributions

AK conceived the study and performed the background literature review. All three authors reviewed the protocol prior to study initiation. AK and DR were responsible for searching the literature, selecting manuscripts and critically appraising them. AK and DR performed the statistical analysis. All three authors assisted in interpreting the results and writing the final manuscript. All authors have read and approved the final manuscript.

## Acknowledgements

The authors acknowledge Dr Candace Poon, who assisted with the translation of one of the manuscripts.

## Supplementary Material

Additional file 1 **Appendix - OVID Search Strategies**. Click here for file
