# Supplementary material for: Optimal glycemic control in neurocritical care patients: a systematic review and meta-analysis
Source: Crit Care. 2012 Oct 22;16(5):R203. doi: 10.1186/cc11812 (PMC3682305; doi:10.1186/cc11812)
Supplement: Additional file 1 — Appendix - OVID Search Strategies. [file cc11812-S1.DOC]

Appendix – OVID Search Strategies

I. MEDLINE search strategy used to identify randomized controlled trials assessing glycemic control strategies in neurocritical care patients (the EMBASE and COCHRANE search involved minor variations)

1 insulin/ (148510)

2 glucose/ (119799)

3 blood glucose/ (116777)

4 (intensive insulin or glycemic control).mp. [mp=protocol supplementary concept, rare disease supplementary concept, title, original title, abstract, name of substance word, subject heading word, unique identifier] (10832)

5 1 or 2 or 3 or 4 (307073)

6 brain injuries/ (37270)

7 brain edema/ (11392)

8 craniocerebral trauma/ (17997)

9 cerebrovascular trauma/ (67)

10 subarachnoid hemorrhage/ (14717)

11 cerebral hemorrhage/ (25300)

12 intracranial hemorrhage/ (2698)

13 brain ischemia/ (31532)

14 stroke/ (44287)

15 cerebral infarction/ (18445)

16 intracranial embolism/ (2426)

17 intracranial thrombosis/ (1454)

18 spinal cord injuries/ (26275)

19 heart arrest/ (20329)

20 hypoxia, brain/ (6262)

21 anoxia/ (48592)

22 (neurocritical* or neuro-critical* or neurological intensive care or neurologic intensive care or traumatic brain injury or brain trauma or diffuse axonal injury or subdural hematoma or epidural hematoma or intracerebral hemorrhage or cerebrovascular accident or cardiac arrest).mp. [mp=protocol supplementary concept, rare disease supplementary concept, title, original title, abstract, name of substance word, subject heading word, unique identifier] (42748)

23 6 or 7 or 8 or 9 or 10 or 11 or 12 or 13 or 14 or 15 or 16 or 17 or 18 or 19 or 20 or 21 or 22 (288577)

24 clinical trial/ (470103)

25 placebos/ (30721)

26 (trial* or random* or placebo).mp. [mp=protocol supplementary concept, rare disease supplementary concept, title, original title, abstract, name of substance word, subject heading word, unique identifier] (1262062)

27 24 or 25 or 26 (1264755)

28 5 and 23 and 27 (466)

II. MEDLINE search strategy used to identify randomized contolled trials assessing glycemic control strategies in critically ill patients (same strategy used in reference 51; EMBASE and COCHRANE search involved minor variations).

1 intensive care unit/ (29805)

2 limit 1 to yr="2008 -Current" (7179)

3 critical illness/ (13232)

4 coronary care unit/ (3886)

5 postoperative care/ (49305)

6 intensive care/ (13148)

7 (intensive care or ICU or critical care or coronary care or recovery room or par or critical illness or burn unit or critically ill or cardiac care).mp. [mp=protocol supplementary concept, rare disease supplementary concept, title, original title, abstract, name of substance word, subject heading word, unique identifier] (211748)

8 1 or 3 or 4 or 5 or 6 or 7 (258084)

9 insulin/ (148510)

10 antidiabetic agent/ (35303)

11 (intensive insulin or glycemic control or blood glucose or insulin).mp. [mp=protocol supplementary concept, rare disease supplementary concept, title, original title, abstract, name of substance word, subject heading word, unique identifier] (344712)

12 blood glucose/ (116777)

13 9 or 10 or 11 or 12 (355704)

14 (trial* or random* or placebo).mp. [mp=protocol supplementary concept, rare disease supplementary concept, title, original title, abstract, name of substance word, subject heading word, unique identifier] (1262062)

15 placebos/ (30721)

16 clinical trial/ (470103)

17 14 or 15 or 16 (1264755)

18 2 and 8 and 13 and 17 (122)
